# Does menopausal hormone therapy (MHT), exercise or a combination of both, improve pain and function in post-menopausal women with greater trochanteric pain syndrome (GTPS)? A randomised controlled trial

**DOI:** 10.1186/s12905-016-0311-9

**Published:** 2016-06-16

**Authors:** Charlotte Ganderton, Adam Semciw, Jill Cook, Tania Pizzari

**Affiliations:** School of Allied Health (Physiotherapy), College of Science, Health and Engineering, La Trobe University, Bundoora, VIC 3086 Australia; School of Health and Rehabilitation Sciences, University of Queensland, St. Lucia, QLD 4072 Australia

**Keywords:** Hormone therapy, Exercise, Gluteal, Oestrodiol, Post-menopausal, Greater trochanteric pain syndrome, Tendinopathy

## Abstract

**Background:**

Greater trochanteric pain syndrome (GTPS) is pathology in the gluteus medius and minimus tendons and trochanteric bursa that causes debilitating tendon pain and dysfunction, particularly in post-menopausal women. Limited evidence in clinical studies suggests hormone changes after menopause may have a negative effect on tendon. This protocol describes a randomised controlled trial comparing the effectiveness of menopausal hormone therapy (MHT) and exercise therapy in reducing pain and dysfunction associated with GTPS in post-menopausal women.

**Method:**

One hundred and sixteen post-menopausal women will be recruited and randomised to receive one of two exercise programs (sham or targeted intervention exercise) and transdermal creams (MHT cream containing oestradiol 50mcg and norethisterone acetate 140mcg or placebo cream). Interventions will be 12-weeks in duration and outcomes will be examined at baseline, 12-weeks and 52-weeks. The primary outcome measure will be the VISA-G questionnaire and secondary outcomes measures will include three hip pain and function questionnaires (Hip dysfunction and Osteoarthritis Outcome Score, Oxford Hip Score, Lateral Hip Pain questionnaire), a global change in symptom questionnaire (using a 15-point Likert scale) and a quality of life measure (AQoL-8D questionnaire). Data will be analysed using the intention to treat principle.

**Discussion:**

This study is the first randomised controlled trial to compare the effectiveness of menopausal hormone therapy therapy alone, and with the combination of exercise therapy, to treat pain and dysfunction associated with GTPS. This study has been pragmatically designed to ensure that the interventions in this study can be integrated into policy and clinical practice if found to be effective in the treatment of GTPS in post-menopausal women. If successful, there is potential for this treatment regimen to be explored in future studies of other persistent tendon conditions in the post-menopausal population.

**Trial registration:**

Australian New Zealand Clinical Trials Registry ACTRN12614001157662 Registered 31 October 2014.

**Electronic supplementary material:**

The online version of this article (doi:10.1186/s12905-016-0311-9) contains supplementary material, which is available to authorized users.

## Background

Greater trochanteric pain syndrome (GTPS) is an overarching term used to describe a degenerative condition of the gluteus medius and minimus tendons (gluteal tendinopathy) and trochanteric bursa (trochanteric bursitis). The condition is characterised by intermittent or continuous pain at, or around, the greater trochanter of the femur [1], often with long-term pain and disability. Greater trochanteric pain syndrome most commonly affects post-menopausal women between 45 and 63 years old [2] and is severely debilitating, resulting in limited activity, quality of life, employment and capacity to exercise. The reported incidence of GTPS is 1.8 per 1000 patients per year in primary care [2] and prevalence rates of 23.5 % in women at risk of knee osteoarthritis [3] and 54 % in recipients of renal transplants [4]. These statistics likely underestimate the prevalence of GTPS since misdiagnosis and lack of recognition of GTPS are common.

The increased prevalence of GTPS in post-menopausal women [2] suggests that deficits in female sex hormone may be implicated in the condition. A number of risk factors for tendinopathy are associated with levels of circulating female sex hormones - advancing age, female gender and body composition [5, 6]. In pre-menopausal women, the likelihood of developing lower limb tendinopathy is lower than that of age matched men [7]. In post-menopausal women, as oestrogen levels decline, collagen production declines, tendon becomes thinner [8] and rates of tendon pathology and the likelihood of tendon rupture increases [9]. It is known that supplemental oestrogen is beneficial for increasing skeletal muscle strength [10], reducing fractures [11], preserving bone mass [12] and preventing a decline in the collagen content of skin [13]. It is feasible that supplemental oestrogen in the post-menopausal population may also preserve tendon collagen [8] and decrease tendon abnormality [6].

Despite this theory, current knowledge of the physiological effect of female sex hormone on tendon is limited [14–16], with some evidence of increased type 1 collagen upregulation and turnover [17–19], increased fibroblast proliferation [20, 21] and inhibition of pro-inflammatory cytokines [18, 22]. Animal studies also point to the upregulation of relaxin receptors and a resultant effect on the extracellular matrix of the tendon [23], however the literature is limited, inconsistent and generally of poor quality. A small number of observational studies [6, 17, 19, 22, 24–26] and one poorly designed randomised controlled crossover study [18] have investigated the effects of hormone therapy on the Achilles and patellar tendon. None of these studies examined patient reported outcome measures (pain, function, quality of life) to evaluate the effects of such interventions. As yet, there are no studies looking at the effects of menopausal hormone therapy (MHT) on gluteal tendon and no rigorous prospective RCT’s have been published in any other tendon. In addition, the optimal dosage, duration and method of administration of MHT to affect molecular, mechanical, and morphological tendon outcomes are unknown.

Traditionally, physiotherapy and exercise are first line interventions in the clinical management of tendinopathy [27–32] with any form of injection (e.g. cortisone, platelet rich plasma or autologous blood) and operative interventions offered subsequent to conservative tendon management [33, 34]. However, for gluteal tendon conditions, an abundance of research exists for both injection and operative methods and little exists for conservative treatment options [35]. If like other lower limb tendons, the gluteal tendons are responsive to load, then a similar treatment algorithm used in that of the patellar and Achilles tendon [36], should be effective in reducing pain and dysfunction.

The most recent systematic review on the effects of treatment of gluteal tendinopathy concluded that there was a need to conduct further randomised controlled trials [35]. Treatment interventions investigated included shockwave therapy, ‘home training’, corticosteroid injections and operative procedures (arthroscopic and open bursectomy, ilio-tibial band release, open bursectomy, tendon reattachment, trochanteric reduction osteotomy and endoscopic repair of gluteus medius tears). Only 1 out of the 14 included studies used exercise as an intervention [37]. Rompe et al. [37] compared a home training program involving progressive slow repetitive exercise (piriformis and ilio-tibial band stretching, straight leg raise, wall squat with ball and gluteal strengthening) with shock wave therapy and corticosteroid injection [37]. Exercise therapy was found to be less superior to corticosteroid injection at 1 month follow up but more superior at the 15 month follow up, indicating the longer-term benefits of exercise therapy. This response is similar to that seen in other tendons [38–40]. This review demonstrates that the role of exercise therapy in the management of greater trochanteric pain syndrome needs further investigation.

The proposed study will investigate two feasible management options for post-menopausal women with GTPS - hormone supplementation and exercise. The aim of this study is to investigate the effect of menopausal hormone therapy and exercise on pain and function in post-menopausal women with GTPS. We hypothesise that supplemental hormones and exercise may be beneficial for reducing pain and dysfunction measured by a clinical significant change in VISA-G scores and a significant improvement in quality of life measures. If successful, there is potential for this treatment regimen to be explored in future studies of other persistent tendon conditions such as Achilles, rotator cuff and lateral elbow tendinopathy in the post-menopausal population.

## Methods

The trial has been registered on the Australian New Zealand Clinical Trials Registry (ACTRN12614001157662) and has a Universal Trial Number (U1111-1160-2743). Protocol modifications will be approved by the ethics committee and communicated via the ANZCTR.

### Ethical approval and consent

The trial has received ethical approval from the La Trobe University Human Ethics Committee (number 14-055). Prior to enrolment, all participants will provide written informed consent to participate in the study, and for the publication of this manuscript (Additional file 1). Ethical standards will adhere to the National Health and Medical Research Council (NHMRC) National Statement [43] and the World Medical Association’s Declaration of Helsinki [44].

### Design

**Fig. 1:**
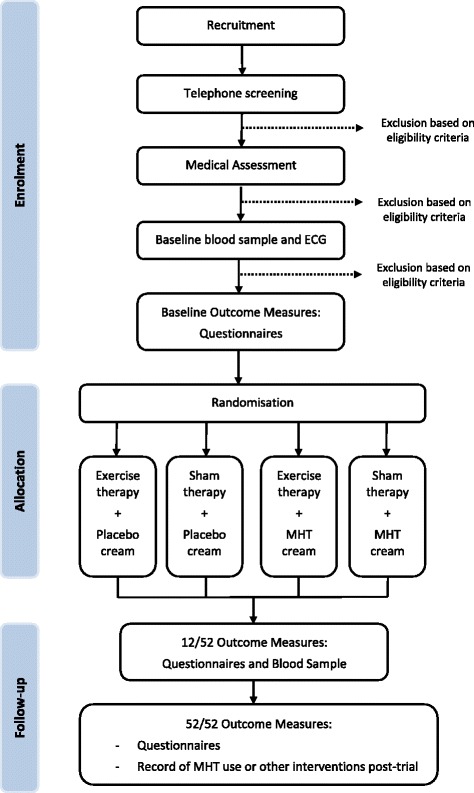
Gluteal La Trobe Trial (GLoBE) Profile

This study is a randomised 2 x 2 factorial trial with a 12-week intervention period and outcomes measured at baseline, 12-weeks and 52-weeks (Fig. 1). Reporting of this study will be formatted according to the Consolidated Standards of Reporting Trials (CONSORT) 2010 statement [45, 46] and incorporates the Standard Protocol Items: Recommendation for Interventional Trials (SPIRIT) [41].

### Participants

#### Recruitment

Post-menopausal women with GTPS will be recruited through medical practitioners and healthcare professionals. Further advertising on community noticeboards, social media, Melbourne radio, newspapers and the Gumtree advertising service will also be conducted. Interested volunteers will contact the chief investigator (CG) via phone or email and will be screened for eligibility (Table 1).

**Table 1:** Eligibility criteria

Inclusion Criteria	Exclusion Criteria
• Post-menopausal women, >52 weeks of amenorrhea and a serum oestradiol of 0-120pmol/L and an FSH of >20 IU/L • Lateral hip pain reproduction in 3 of 5 pain provocation tests (Trendelenburg test, palpation of the greater trochanter, FABER, resisted external derotation test, modified resisted external derotation test) • Have sufficient English skills to be able to read and understand the information and consent form due to the risks involved with participating in the study.	• Known adverse reaction to any form of hormone therapy • Use of any form of female hormone supplementation within the last 12 weeks • Display high risk factors for deep vein thrombosis (DVT) using the Wells score system [85] and/or pulmonary embolism (PE). • Current smoker • History of stroke, severe menstrual migraine, endometrial sarcoma, breast cancer, severe hyperlipidaemia, chronic liver disease, thyroid disease, lupus, hysterectomy and oophorectomy • HbA1c >8 % • Current cholecystitis, fibroids or undiagnosed abnormal uterine bleeding • Platelet-rich plasma (PRP), analogous blood injection (ABI) or corticosteroid injection into the hip region in the last 12 weeks • History of hip trauma or surgery on the affected side • Any other musculoskeletal, neurological and cardiorespiratory condition/s affecting their ability to participate in the study.

### Study procedure

#### Telephone screening

Potential participants will initially be screened via telephone to exclude those with obvious medical risk factors to MHT (Table 1). Further exclusion is warranted if the potential participant has received hormone therapy supplementation or invasive procedures (including injection) around the hip in the previous 12 weeks. If no obvious exclusion criteria exist, the participant will be referred for medical assessment.

#### Medical assessment

Participants found to be eligible after the phone screening will be invited to attend a medical examination by a sports physician. Upon presentation to their medical examination, participants will again be educated on the nature of the trial and asked to provide informed consent. Potential participants will be advised at that point that inclusion in the trial is on the provision that their medical history, clinical examination, blood samples and electrocardiograph (ECG) meet inclusion criteria.

The medical consultation will involve a full medical history to determine risk factors that would contraindicate the use hormone supplementation, and perform a clinical examination for the presence of greater trochanteric pain. Medical history will detail the history of presenting condition, family history, past medical history, social history (demographics, occupation, and recreation/activity participation), medication usage, substance use/abuse (alcohol, recreational drugs, smoker status) and allergies.

Clinical assessment to assess GTPS will involve five clinical pain provocation tests. Tests will include the Trendelenburg test [41], palpation of the greater trochanter [42], the Patrick-Faber test [43], resisted external de-rotation test [41] and modified external de-rotation test (increased hip adduction moment). Participants are asked if testing reproduces reported symptoms or pain, the location of the symptoms and intensity of pain on a 0–10 Visual Analogue Scale (VAS). A positive test is defined as a spontaneous reproduction of the participant’s lateral hip pain. To be eligible for the trial, clinical testing must reproduce pain in 3 of 5 pain provocation tests.

Where a participant meets inclusion criteria and has not had a platelet-rich plasma (PRP), analogous blood injection (ABI) or corticosteroid injection in the hip region in the last 3 months, but is excluded from the hormone intervention on medical grounds, they will be randomised into a single arm (intervention exercise or sham exercise).

#### Blood sampling and electrocardiography

Participants who remain eligible for the study following medical consult, will be referred for blood tests and an electrocardiograph to confirm eligibility in the trial. Blood tests will examine lipid levels (fasting cholesterol, triglycerides, high density lipoprotein: HDL, low density lipoprotein: LDL), liver function (LFT), diabetes indicators (glucose, HbA1c), iron (Fe) studies and thyroid function (TFT). Serum levels of follicle stimulating hormone (FSH) and oestradiol will also be recorded as a baseline measure and compared to levels post pharmacological intervention. A 12 lead echocardiograph (ECG) will be undertaken to monitor if any arrhythmia or irregularities are present. Indicators for inclusion or exclusion of a participant will be made on clinical judgement by the treating physician, taking into account all individual tests results and their normative values.

Once deemed eligible for the trial, participants will be randomly allocated into an exercise group and a transdermal cream group. A participant may be randomised into one of the following 12-week interventions: (i) exercise therapy, placebo cream; (ii) sham exercise, MHT cream; (iii) sham exercise, placebo cream or (iv) exercise therapy, MHT cream.

### Sample size and power analysis

The sample size required for this RCT is 100 participants (25 in each group) to detect a minimal clinically important difference of 10 points on the VISA-G outcome measure, assuming a standard deviation of 13 (Alpha of 0.05 and a power of 80 %) [44]. To account for potential drop-out, we will recruit 116 participants (29 per group).

### Randomisation, allocation and blinding

A block randomisation schedule will be generated using a web based randomisation program (https://www.randomizer.org/) with the sequence tranferred onto a computer spreadsheet by a researcher external to the trial investigators, who will have no contact with participants throughout the duration of the trial. This external researcher will complete concealed allocation of participants to groups in accordance with the randomisation schedule. All data collected will be de-identified and only made accessible to the study investigators.

Participants will be blinded to allocation for both the exercise therapy and hormone therapy group. Placebo creams and sham exercise groups, may increase the success of participant blinding [46]. The success of blinding will be formally measured during week 1 of the trial by asking participants to indicate which group for both interventions they believe they are allocated to. It is important to assess the success of blinding to determine if protection against participant expectation effects is maintained [46]. The treating physiotherapists will be blind to cream type, but due to the nature of the intervention, cannot be blinded to exercise group. Detailed training of physiotherapists will be provided to ensure equivalent provision of care and motivation for both groups [47]. Unblinding of a participant and trial investigators to cream allocation will only be permissable in the unlikely event of an adverse reaction requiring medical team involvement.

Data analysts and outcome measure assessors will be blinded to group allocation. This will be achieved through direct communication from the external researcher preparing the randomisation schedule to the trial physiotherapist implementing the exercise intervention and the pharmacist implementing the hormone cream intervention.

### Interventions

All participants will be educated on the nature of their pathology, the rationale for exercise treatment, the importance of adherence to their home program as well as activities to avoid (ascending and descending stairs, sitting in low chairs, lying on hip during sleep). They will be provided an information booklet that includes this information. The booklet details pain relieving postural strategies (standing, sitting and lying/sleeping) to adopt during the 12-week intervention. Participants may apply an ice pack to the lateral hip region at any time; are encouraged to stay active within their pain limits and to continue their normal pain medication regime during the 12-week intervention. Both medication use and ice application will be recorded throughout the trial in the participant’s exercise/transdermal cream application diary. All participants are able to continue normal daily activities but will be asked to identify any medication usage for their tendinopathy (e.g. NSAIDs) and cease any other physical treatment (physiotherapy, etc.).

#### Exercise therapy

Both exercise therapy groups will complete a 12-week exercise program prescribed by a physiotherapist. Participants in both groups will attend a physiotherapy consultation at baseline, 4-weeks, 8-weeks and 12-weeks post randomisation for progression of their exercise program and monitoring of adherence. A booklet that features photos and explanations of each exercise will be given to each participant for further reference and participants are invited to contact their physiotherapist at any time during the 12 week intervention if they have queries about their cream or exercise program. Before, during and after the 12 week program, participants are invited to contact the researchers with any questions about the trial. Two cycles of the exercise program will be completed twice daily (AM and PM). Both programs should take approximately 15 min to complete.

#### Gluteal La Trobe trial (GLoBE) protocol/intervention exercise therapy

Participants randomised into the intervention exercise group will be instructed in the GLoBE protocol, a gluteal tendon exercise program designed by clinicians who are expert in treating tendon pathology. Although numerous rehabilitation exercises (eccentric [32, 48], concentric [30, 49] and isometric [50]) have been described for the management of Achilles and patellar tendinopathy, there is little evidence on effective exercise therapy in the rehabilitation of GTPS [37]. There is evidence that the use of isometric exercises may reduce tendon pain in the initial stages of rehabilitation [50] and allow the continuation of sporting activities [51]. This pain relief may provide a window of opportunity for lower limb strengthening with isotonic exercise. The effectiveness of isometric exercises is yet to be validated in the GTPS population. The GLoBE protocol largely focuses on isometric loading of the gluteus medius and minimus, and strengthening of the kinetic chain – quadriceps and calf muscle groups. It is a graduated exercise program where participants are able to progress through the stages in each subsection of gluteal, quadriceps and calf strengthening. The gluteal strengthening commences with a hip hitch in standing, whereby the participant, holding onto a wall or chair for balance, hitches their unaffected leg up off the ground (approximately 1 cm) whilst keeping their knee in full extension, causing the contralateral/affected gluteal tendons to be loaded out of a compressed position. This hip hitch is then integrated into dynamic exercises by adding a toe taps or hip swings as progressions. The most advanced exercise in the gluteal series, is a single leg wall squat. Quadriceps strengthening commences with a double leg ¼ squat and progresses to a ½ squat, sit to stand exercise and then to step ups. Calf strengthening involves double leg calf raises, calf raises with toe taps and progresses onto single leg calf raises.

#### Sham exercise program

The control group will receive a sham exercise program not suitable for the rehabilitation of gluteal tendons, instructed by a physiotherapist. Like the GLoBE protocol, the program is completed as a cyclic three-stage approach, where participants are able to progress through each subsection of sham gluteal, quadriceps and calf strengthening. The sham program involves exercises for the kinetic chain in an unloaded environment (sitting) and thus, does not therapeutically load the gluteal tendons.

### Physiotherapy treatment integrity

A variety of methods will be used to ensure the integrity of the exercise intervention is maintained. All treating physiotherapists will have a minimum of 2 years clinical experience in private musculoskeletal physiotherapy practice and will undertake a compulsory 1 day training course. This will incorporate extensive education about GTPS, a step-by-step approach to the two exercise interventions and guidelines on trial reporting (participant adherence, adverse events, and clinical note taking). The education session will include group discussions and portions of experiential learning such as patient role play and formal clinical mentoring as outlined by Main et al., [47] for training physiotherapists delivering interventions. Regular phone and email contact with the researchers and provision of a comprehensive treatment manual will aim to maximise therapist adherence to the protocol. Additionally, a large emphasis will be placed on the importance of equal service provision and standardisation of enthusiasm of both exercise protocols [52, 53].

Physiotherapists will use structured electronic recording forms for sham and intervention exercise programs. These will include a series of check boxes to prompt the clinician through each stage of the program and to ensure standardisation of treatment intervention. Additionally, the physiotherapist will be instructed to record exercises prescribed, adherence, use of pain or NSAID medications and/or ice application and any adverse events. These measures adhere to the recommended requirements for ensuring treatment fidelity [47]. Clinical notes will be audited by research staff at 4-weeks and 12-weeks to ensure that all documentation is standardised, legible and complete.

### Transdermal cream

All participants will be required to apply 1 g of the compounded transdermal cream daily to thin-skin areas of the body to aid absorption and rapid distribution of active ingredients. Cream application sites will be rotated every 5 days – alternating between the inner right wrist and the inner left wrist. Rotation of sites every 5 days will reduce the likelihood of up-regulation of receptors [54]; skin sensitivity by constant application to the same area and undesired build-up and delayed uptake of active ingredients [55]. Participants will be advised to not to rub in too hard, but simply to spread the gel over the skin as thinly as possibly to optimise absorption [56].

Both the placebo and intervention transdermal creams will be applied using a metered dose pump dispenser manufactured by Medisca, a Therapeutic Goods Administration (TGA) registered supplier of pharmacy compounding products. This dispenser accurately delivers the required dose (1 ml) in one pump (mean error = 0.056 ml, relative SD = 4.4 %) [57]. Both intervention and placebo groups will receive an identical instruction sheet for the application of the cream. The method of application will also be reinforced verbally by the physiotherapist in the first exercise treatment session. Any significant or severe adverse reactions will immediately be referred to a medical practitioner or emergency department for assessment.

#### Menopausal hormone therapy transdermal cream

The MHT cream is based on the active ingredients used in the Estalis Continuous transdermal patch [58]. Cream application will involve transdermal delivery of supplementary oestradiol (50mcg) and norethindrone acetate (NETA) (140mcg), a progestin, in a VersaBase® for 12 weeks in duration. This is likely to increase serum levels of E_2_, with an aim to reach an optimum level of 295–550pmol/L [54]. The progestin component provides essential protection of the uterus to reduce the risk of endometrial hyperplasia [59].

#### Placebo transdermal cream

The placebo transdermal cream, applied for a duration of 12 weeks, will be an inert aqueous transdermal cream that is identical in colour, texture and consistency to that of the MHT cream but without the active ingredients.

### Baseline assessments

At baseline, a thorough medical history will be taken, time-interval since menopause documented and current medication use and dosage will be recorded. Height and weight will be measured using a stadiometer and digital scales, respectively, and body mass index will be calculated as weight (kg)/height (m)^2^. In addition, six questionnaires will be administered to monitor pain, function and quality of life.

### Outcome measures

#### Primary outcome measures

The primary outcome measure will be the VISA-G questionnaire, a gluteal tendon outcome measure that quantifies pain with tendon loading, with a higher score representing less pain and dysfunction. This questionnaire contains a visual analogue score for pain, four questions related to pain, one question related to difficulty with moving after sitting and two activity related questions [60]. The VISA-G was found to have a test retest reliability of ICC_2,1_ (95 % CI) of 0.83 (0.64 to 0.78) [60]. Internal consistency was high with a Cronbach’s Alpha of 0.81. Construct validity was demonstrated: the VISA-G measures different constructs than both the Harris Hip Score (HHS) [61] and the Oswestry Disability Index (ODI) [62] (Spearmans rho: 0.02 and 0.02 respectively). The VISA-G did not demonstrate any floor or ceiling effect in symptomatic participants. In the current study, the VISA-G will be documented at baseline, 12-weeks and 52-weeks.

#### Secondary outcome measures

Secondary outcome measures will include five questionnaires to assess pain and dysfunction. The following questionnaires will be administered at baseline, 12-weeks and 52-weeks: Oxford Hip Score (OHS) [63], global rating of change questionnaire, Assessment of Quality of Life (AQoL), Hip dysfunction and Osteoarthritis Outcome Score (HOOS) [64] and the Lateral hip Pain Questionnaire (unpublished, University of Queensland, Australia).

### Failure to improve

Participants will be offered cross over to both active interventions if their global rating of change score is below 0 (worsened symptoms) at 12 weeks. If in GLoBE exercise and placebo cream, they will cross over to intervention cream and continue GLoBE exercise. If in sham exercise and placebo cream, they will cross over to GLoBE exercise and intervention cream, if in sham exercise and intervention cream, they will cross over to GLoBE exercise and continue intervention cream once cleared to do so by study medical practitioners. Results for this group will be analysed and presented separately.

### Trial follow-up

At 52-weeks, participants will be asked to complete the six questionnaires again, and asked to specify if they continued any hormone therapy interventions or had any form of injection (cortisone, protein-rich-plasma or autologous blood injections) or surgical procedure post-trial.

### Success of blinding

To assess the effectiveness of blinding and to ensure participant expectation effects were protected, participants will be asked at one-week post intervention if they know which group they have been randomised to with the following two questions: “This trial compares the effect of two exercise programs, a new program and a standard program. Are you aware of which exercise program you received?”; “This trial compares the effect of two transdermal creams, a hormone therapy cream and a placebo cream. Are you aware of which transdermal cream you have received?” For both questions, the participant can answer with “yes”, “no” or “unsure”. If the participant answers “yes” the participant will be further questioned “which exercise program/cream do you think you received?” and “why” [46].

### Adverse events

Complications and adverse events associated with the intervention are unlikely, however, minor, significant and severe adverse events for this trial are based on the MIMS Australia Estalis Continuous (oestradiol/NETA: norethisterone acetate) consumer information sheet [58]. All adverse events will be recorded in the clinical notes, significant events referred to the treating medical practitioners and severe events referred to the emergency department. Significant and severe adverse events will be reported to the La Trobe University Human Ethics Committee, Faculty of Health Sciences using a standardised form. A participant has the right to withdraw from the study at any time.

*Minor Adverse Event:* Refers to any symptom or event reported by the participant that may be potentially related to the intervention. This will include:

- irregular vaginal bleeding or spotting
- tender, painful or swollen breasts
- menstruation-like pain
- redness, irritation or itching at the site/s of cream application
- skin rash
- vaginal itching, inflammation or fluid discharge
- swelling of the lower legs, ankles, fingers or abdomen due to fluid retention
- nausea, abdominal cramps, vomiting, heartburn, wind, diarrhoea
- headache, migraine
- rise in blood pressure
- weakness or dizziness
- depression, nervousness, rapid changes in mood, difficulty sleeping
- back pain
- change in sex drive
- weight gain
- acne, itchy or dry skin, skin discolouration
- darkening of the skin particularly on the face or abdomen (chloasma)
- hair loss

*Significant Adverse Event:* Any symptom or event potentially related to the intervention that interrupts the participant’s ability to continue with the RCT intervention, or requires the participant to be referred to a medical practitioner. This will include:

- heavy vaginal bleeding
- pain or tenderness in the abdomen, which may be accompanied by fever, loss of appetite, nausea and vomiting
- a yellow colour to the skin or eyes, itching, dark coloured urine or light coloured bowel motions

*Severe Adverse Event:* Any symptom or event potentially related to the intervention that results in admission to the hospital, or permanent disability, or is life threatening. This will include:

- swelling of the face, lips, tongue, the area around the eyes or other part of the body
- rash, itching, hives, breathlessness or difficult breathing, wheezing or coughing
- light-headedness, dizziness, changes in levels of consciousness, hypotension, with or without mild generalized itching, skin reddening
- signs that blood clots may have formed, such as sudden severe headache, sudden loss of coordination, blurred vision or sudden loss of vision, slurred speech, numbness or tingling in an arm or leg, painful swelling in the calves or thighs, chest pain, difficulty breathing, coughing blood

### Statistical analysis

Statistical analysis will use intention to treat principles and per protocol analysis. A mixed model ANCOVA will be undertaken, adjusting for baseline values of the dependent variables to determine the difference between groups for primary and secondary outcomes. In addition, a responder analysis will be undertaken to determine clinical significance [65]. Participants who achieve an increase in VISA-G scores greater than the minimal detectable change score (10 points) [60] will be categorised as responders. The difference between the proportions of responders and non-responders in the four groups will then be analysed with risk ratios, risk differences and numbers needed to treat [53]. Participants randomised into the single exercise arm will be analysed separately. Where data is missing, baseline data will be carried forward.

## Discussion

This study will be the first randomised controlled trial to compare the effectiveness of MHT alone, and with the combination of exercise therapy, to treat pain and dysfunction associated with GTPS. This study has been pragmatically designed to ensure that interventions implemented in this study can be integrated into policy and clinical practice if found to be effective in the treatment of GTPS in post-menopausal women. It aims to minimise bias through methodological features of randomisation, blinding where possible, concealed allocation and intention to treat analysis.

There are risks with any study involving pharmacological interventions, however considerable efforts have been made to minimise these. A transdermal method of administrating hormones has been chosen as it combines an efficacious use of oestrogen replacement with the safest route of administration [66]. Additionally it is the gold standard for menopausal hormone replacement therapy, as it has a lower risk of venous thromboembolism than oral administration [67]; it is easily formulated and titrated; and has an additional safety benefit of avoiding the hepatic ‘first pass’ effect associated with oral administration, thus avoiding changes in clotting factors and sex hormone-binding globulin [68].

The validity of GTPS as a clinical entity has been determined using convergence of validity principles, previously described by George and Delitto [69], and diagnostic criteria based on by Fearon et al. [70]. The convergence of validity principles involves gathering evidence from multiple sources with differing methodologies to determine if the underlying constructs are of similar meaning. Three main principles have been selected for the current study: expert opinion; biological plausibility; and diagnostic tests/strategies for minimising false positive diagnoses.

The validity of GTPS as a clinical entity is supported by research on biological plausibility. Pathological features of gluteus medius and minimus tendinopathy have been likened to that of supraspinatus tendinopathy and subacromial bursitis of the shoulder [1, 71–73]. Anatomically, the gluteus medius tendons inserts onto the lateral and posterior-superior portion of the greater trochanter [74], and the gluteus minimus onto the anterior portion of the greater trochanter and acts as a femoral head stabiliser through hip joint range [75]. The positioning of these tendons is such that loss of the lateral stability mechanism (frontal plane femoropelvic alignment and medio-lateral stability in standing) from hip abductor weakness [76], can cause compression of these structures and reproduction of lateral hip pain. Similarly, pain reproduction with passive elongation of the involved tendons or active contraction of these same tendons is reported as a clinical feature of gluteal tendinopathy/GTPS [42]. Clinical tests used to assess for GTPS in the trial are likely to reveal symptoms associated with one or both of these mechanisms. Additionally, common subjective clinical findings such as difficulty lying on their side at night [77, 78] and pain with weight bearing activities [1, 78, 79] will be identified using the VISA-G questionnaire.

Due to the difficulties in diagnosing GTPS, there are aspects of our diagnostic criteria that are likely to improve diagnostic accuracy and minimise false positive diagnoses. Tendon experts recommend a battery of tests be used for clinical assessment and interpretation of hip abductor function [76]. This study will only include participants if they score <80 on the VISA-G questionnaire (a measure of pain and dysfunction associated with the condition) and have reproduction of lateral hip pain on a minimum of 3/5 diagnostic clinical tests.

Based on the convergence of this quantitative research, as well as the expertise of one of the authors (JC) who is a world-renowned tendon expert, with over 30 years of clinical experience in the area of tendon structure, function and dysfunction, the diagnostic criteria used to identify GTPS in this study have acceptable validity.

Development of the gluteal tendon rehabilitation program is based on expert opinion and guided by management strategies of other lower limb tendons. Commencing the program with gluteal isometrics is used as an initial pain relieving strategy [50] prior to loading with internal perturbations (toe taps and hip swing). All gluteal exercises are performed avoiding compression of the gluteal tendons (no hip adduction/crossing midline), since compression is thought to induce tendon pathology [80] by altering mechanotransduction and causing fibrocartilage formation [81]. Additionally, activities involving hip adduction including side-lying in bed [77, 78], and weight-bearing activities (stair climbing, rising from a chair) have been associated in lateral hip pain [1, 78, 79]. In this program, all exercises aim at loading the gluteal tendon occur on a single leg as double leg exercises allow compensation with the other leg [82] and inefficient tendon loading. Furthermore, this program focuses on rehabilitation of the kinetic chain - sequenced physiologic muscle activations that allow performance of an integrated biomechanical task [83]. This has been recommended to ensure complete restoration of lower leg muscle-tendon function [84]. The sham exercise program, although activating the same muscle groups as the intervention, clinically, these exercises are not aimed at strengthening muscle groups or loading the gluteal tendons.

Study results will be disseminated to the wider health network by journal publication, and reported back to participants via email. The findings of this study will help determine the effectiveness of menopausal hormone therapy and exercise therapy in post-menopausal women with GTPS. Additionally it will identify if an exercise program targeted at loading of the gluteal tendons in addition to kinetic chain strengthening is more superior to that of a low load lower limb sham exercise program. The results of this study aim to help guide medical and allied health practitioners to make an informed decision on the management of GTPS.

## Trial status

Ongoing.

## Abbreviations

AQoL, assessment of quality of life; Fe, iron; FSH; follicle stimulating hormone; GTPS, greater trochanteric pain syndrome; HbA1c, haemoglobin A1c; HDL, high density lipoprotein; HHS, harris hip score; HOOS, hip dysfunction and osteoarthritis outcome score; LDL, low density lipoprotein; LFT, liver function test; MHT, menopausal hormone therapy; ODI, oswestry disability index; OHS, oxford hip score; TFT, thyroid function tests; VISA-G; victorian institute of sport Australia – gluteal questionnaire

## Additional file

Additional file 1: Participant Consent Form. (DOCX 58 kb)
