# Supplementary material for: Does menopausal hormone therapy (MHT), exercise or a combination of both, improve pain and function in post-menopausal women with greater trochanteric pain syndrome (GTPS)? A randomised controlled trial
Source: BMC Womens Health. 2016 Jun 16;16:32. doi: 10.1186/s12905-016-0311-9 (PMC4910216; doi:10.1186/s12905-016-0311-9)
Supplement: Additional file 1: — Participant Consent Form. (DOCX 58 kb) [file 12905_2016_311_MOESM1_ESM.docx]

**La Trobe University Human Ethics Committee Participant Consent Form**

**Project Title:** Does female sex hormone supplementation, exercise or a combination of both, improve pain and function in post-menopausal women with greater trochanteric pain syndrome (GTPS)? A randomised controlled trial. UHEC 14-055

Investigator no.1 **Ms Charlotte Ganderton** (PhD candidate)

Investigator no.2 **Dr Tania Pizzari**

Investigator no.3 **Dr Adam Semciw**

Investigator no.4 **Professor Jill Cook**

I ­­­­­­­­­­­­­____________________________ have read and understood the **participant information statement and consent form; understood all potential risks associated with my participation**, and any questions I have asked have been answered to my satisfaction. I understand that even though I agree to be involved in this project, I can withdraw from the study at any time, up to four weeks following the completion of my participation in the research. Further, in withdrawing from the study, I can request that no information from my involvement be used. I agree that research data provided by me or with my permission during the project may be included in a thesis, presented at conferences and published in journals on the condition that neither my name nor any other identifying information is used.

| I am willing to have photographs and/ or videos taken during the testing session and consent for these images or videos to be used solely for education and research purposes at physiotherapy schools at other universities in Australia and when presentations are made at conferences / workshops in National and International Settings. | Yes | No |
| --- | --- | --- |
|  |  |  |

| Last Name: | Given Name: |
| --- | --- |
| DOB: | Contact Phone number: |
| Address: |  |
| Participant Signature: | Date: |
| Witness name: |  |
| Witness Signature: | Date: |

**Name and phone number of contact person in case of an emergency:**

| Name: | Phone: |
| --- | --- |
| Family Doctor: | Phone: |
